# Celebrating 10 Years: Insights From CMGH’s Editorial Fellows

**DOI:** 10.1016/j.jcmgh.2025.101603

**Published:** 2025-08-23

**Authors:** Kole H. Buckley, Lin Y. Hung

**Affiliations:** Department of Gastroenterology and Hepatology, Perelman School of Medicine, University of Pennsylvania, Philadelphia, Pennsylvania; NYU Pain Research Center, Department of Molecular Pathobiology, College of Dentistry, New York, New York

Our time as editorial fellows at *Cellular and Molecular Gastroenterology and Hepatology* (*CMGH*) has been an enriching and profoundly positive experience. Prior to this fellowship, our scientific publishing experience had been limited to submitting articles. Having the opportunity to step behind the curtain of scientific publishing has offered invaluable insights, fundamentally enhancing our understanding of what goes into shaping impactful research dissemination, and how to do so in an ethical and efficacious manner.

Working side by side with co-editors-in-chief Michele Battle and Jonathan Katz, one of the most significant takeaways has been the refinement of our ability to critically evaluate scientific papers. We have learned to meticulously assess manuscripts not just for their content, but for their rigor, novelty, and potential impact to advance a given field. This goes beyond simply understanding the science. It involves discerning the methodological soundness, the originality of the findings, and the true significance of the work within the broader landscape of gastroenterology and hepatology basic science research. Furthermore, this improved analytical lens has also sharpened our skills in summarizing the main points of a manuscript concisely and effectively, enabling clearer communication with the board of editors in our weekly meetings. This is undoubtably a valuable skill to a young scientist, even outside of publishing.

The fellowship has provided a comprehensive look at the entire editorial process, from initial submission to final publication. Participating in the intricate dance between authors, reviewers, and editors has fostered a deeper appreciation for the effort involved in upholding scientific standards. This includes understanding the critical importance of identifying and addressing potential fraud or malpractice in order to maintain research integrity, particularly in today’s world.

Environmentally, the *CMGH* board of editors and supporting staff have been terrific. As editorial fellows, we have been treated with the same respect afforded to all sitting board members and *CMGH* staff. Venturing beyond our comfort zones as young investigators, we were able to express our opinions, insights, and expertise in an environment that fostered kindness, acceptance, and growth.

Our time as editorial fellows at *CMGH* will always be remembered and cherished. One indelible experience was a board meeting discussion highlighting that overly negative, critical, and even malicious reviewer comments hinder scientific progress. In an era where science faces high scrutiny, politicization, and vulnerability, it is paramount that we forge ahead united, fostering a supportive and unwavering community of resilient scientists. For these reasons, *CMGH* is committed to driving initiatives that champion diversity, equity, inclusion, and mentorship.

Overall, this fellowship has been more than just an educational opportunity. It has been a journey that has profoundly enhanced our skills in a way that the laboratory bench simply cannot and provided a newfound perspective on the vital role of scientific journals in advancing knowledge. This experience as editorial fellows has transformed the way we critically evaluate our own research. It has also created new opportunities for networking and mentorship that are beyond our normal reach as young investigators.

Our year with *CMGH* will undoubtedly be one of our most memorable accomplishments as junior researchers. As *CMGH* celebrates its 10-year anniversary, we reflect on the community of researchers and editorial staff, past and present, who have contributed to the journal’s current position as the top basic science journal in the field. We are very fortunate to be this past year’s editorial fellows and have the privilege of celebrating such a milestone year. We have all played an essential role in powering and growing *CMGH* to where it is today. We look forward to seeing the journal reach new heights of scientific excellence.

